# First Record of *Ixodes ariadnae* (Acari: Ixodidae) in Slovakia

**DOI:** 10.3390/ani16030391

**Published:** 2026-01-27

**Authors:** Jakub Lipinský, Patrícia Petroušková, Monika Drážovská, Emília Vasilová, Miloš Halán, Blažena Hajdová, Anna Ondrejková, Boris Vojtek, Marián Prokeš, Lýdia Gogoľová, Katarína Franková, Ľuboš Korytár

**Affiliations:** 1 Department of Epizootiology, Parasitology and Protection of One Health, University of Veterinary Medicine and Pharmacy in Košice, Komenského 73, 041 81 Košice, Slovakia; jakub.lipinsky@student.uvlf.sk (J.L.); patricia.petrouskova@uvlf.sk (P.P.); monika.drazovska@uvlf.sk (M.D.); milos.halan@uvlf.sk (M.H.); blazena.vargova@uvlf.sk (B.H.); anna.ondrejkova@uvlf.sk (A.O.); boris.vojtek@uvlf.sk (B.V.); marian.prokes@uvlf.sk (M.P.); lydia.gogolova@student.uvlf.sk (L.G.); katarina.frankova@student.uvlf.sk (K.F.); 2 Clinic of Birds, Exotic and Free Living Animals, University of Veterinary Medicine and Pharmacy in Košice, Komenského 73, 041 81 Košice, Slovakia; emilia.vasilova@student.uvlf.sk

**Keywords:** *Ixodes ariadnae*, Slovakia, *Myotis myotis*, tick distribution, bat parasites

## Abstract

Herein we report the first occurrence of *Ixodes ariadnae* in Slovakia. One engorged female was collected from a greater mouse-eared bat (*Myotis myotis*) in the spring of 2025. Identification of the collected *I. ariadnae* specimen was based on morphological characters and confirmed by sequencing *COI* genes and mitochondrial 16S rDNA. Sequences showed > 99–100% similarity to specimens collected in other European countries. This expands the known range of *I. ariadnae* and calls for the further investigation into its host associations, prevalence, and potential role in tick-borne pathogen transmission among European bats.

## Simple Summary

The detection of *Ixodes ariadnae* in Slovakia expands the known distribution of this bat-associated tick species, which has been confirmed in Europe (Hungary, Germany, Belgium, and Turkey) and Asia (Turkey and Japan). To date, 25 hard ticks, including *I. ariadnae*, have been recorded in Slovakia. This paper includes morphological and molecular evidence of one engorged female *I. ariadnae* collected from a greater mouse-eared bat (*Myotis myotis*) in Slovakia. The bat was found exhausted during the spring migration period. As Slovak and Hungarian populations of *M. myotis* move between roosts in both countries, we assumed that the bat was returning from Hungarian wintering grounds where *I. ariadnae* is present.

## 1. Introduction

Bats (Order: Chiroptera) form the second-most species-rich mammalian order with more than 1430 taxa (https://batnames.org/; accessed 20 November 2025) [1]. Bats provide important ecosystem services [2,3]. Moreover, bats are increasingly recognized as ecologically and epidemiologically important species that host a diverse fauna of ectoparasites, including ticks, which serve as vectors for pathogens that are often zoonotic [4,5,6,7]. Consequently, bats and their ectoparasites form a key link in complex host–parasite networks [8,9].

Parasitiform ticks (Acari) are globally distributed ectoparasites of ectothermic and endothermic terrestrial vertebrates [10,11]. The genus *Ixodes* Latreille, 1795, belonging to the family Ixodidae Murray, 1877, includes several taxa that are highly specialized to bats [12].

By 2014, only two species of ixodid ticks closely associated with bats were reported [13]: *Ixodes simplex* Neumann, 1906, and *Ixodes vespertilionis* Koch, 1844. These two species differ in their host preference, with *I. simplex* parasitizing mainly the species *Miniopterus schreibersii* [14], while *I. vespertilionis* has been described mainly in species of the genus *Rhinolophus* [15].

Another bat-associated representative of the family is *Ixodes ariadnae* Hornok, 2014, described as a new species of tick found in caves in Hungary [16]. The host preference of *I. ariadnae* appears to be *Myotis* spp. [17], including *M. alcathoe*, *M. blythii* [16], *M. myotis* [18], *M. bechsteinii*, *M. nattereri*, *M. emarginatus*, *M. daubentonii*, and *M. dasycneme* [19]. Hornok et al. [16] also reported its presence on the host *Plecotus auritus*.

**Figure 1:**
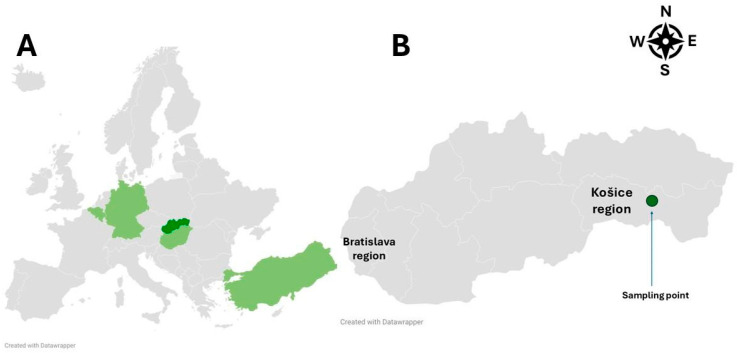
Geographical distribution of *Ixodes ariadnae* in European countries, with Slovakia marked in dark green (**A**) and the location of the first detection in Slovakia marked by a green dot (**B**).

The known geographical distribution of the *Ixodes ariadnae* tick in Europe is currently limited to Hungary, Germany, Belgium, and Turkey (Figure 1A) [18,20,21]. The expansion of ixodid ticks in Europe both towards northern and higher-altitude regions as well as within their original endemic areas is influenced by a variety of environmental and biological factors [22,23].

In this paper, we describe the first detection of *I. ariadnae* in Slovakia. An *I. ariadnae* female was collected from a greater mouse-eared bat (*Myotis myotis*). Prior this report, *I. ariadnae* had not been recorded as part of the tick fauna in Slovakia.

## 2. Materials and Methods

Handling of the greater mouse-eared bat and sample collection was authorized by the Ministry of Environment of the Slovak Republic based on official permit No. 3051/2019-6.3 and its prolongation No. 13625/2023-6.3 granted to selected researchers from the UVMP in Košice, namely A.O., M.P., and Ľ.K., and on permit No. 4801/2023-6.3 granted to field consultants of the Slovak Bat Protection Society, namely Ľ.K.

The infested host, a greater mouse-eared bat (*Myotis myotis*) was admitted at the Clinic of Birds, Exotic and Free Living Animals of the University of Veterinary Medicine and Pharmacy in Košice on 11 April 2025. The bat was identified using an identification key by Dietz C. and Helversen O., “Illustrated identification key to the bats of Europe” (https://www.mammalwatching.com/Palearctic/Otherreports/batkey.pdf, accessed on 23 January 2026). The bat was found unable to fly and heavily exhausted by members of the public in the outdoor area of a housing estate at GPS 48°44′15.4″ N 21°17′05.7″ E (Figure 1B). Subsequently, due to its poor physical condition and exhaustion, the bat was taken into rehabilitation. Despite rehydration therapy and supplementary feeding, the bat died two days after admission. The *Ixodes* sp. was found during the initial clinical examination, transferred to a sterile tube, and determined morphologically.

### 2.1. Morphological Identification

The tick was identified under a trinocular stereozoom microscope Optika SLX–3 (Ponteranica, Italy) and Nikon SZM 1400 (Shinagawa City, Japan) using the morphological key according to Hornok et al. [16]. Photos were taken by a Canon EOS 1100d (Ota City, Japan) and processed. Measurements were taken using Promicra QuickPHOTO software 3.0 with the Deep Focus extension 3.4.

### 2.2. DNA Extraction

After morphological identification of the tick, genomic DNA was isolated using the blackPREP Tick DNA/RNA Kit (IST Innuscreen GmbH, Berlin, Germany) according to the manufacturer’s instructions. DNA was eluted in 75 µL of the elution buffer and stored at −20 °C until analysis. Spectrophotometric measurements (NanoDrop One; Thermo Fisher Scientific, Waltham, MA, USA) were used to determine DNA concentration and purity. Sterile PBS was included as a negative extraction control.

### 2.3. Molecular Analysis and Sequencing

As the initial target for molecular analysis, the cytochrome c oxidase subunit I (*COI*) gene was amplified by a modified PCR as described by Hornok et al. [19]. The PCR was performed using specific primers HCO2198 (5′-TAAACTTCAGGGTGACCAAAAAATCA-3′) and LCO1490 (5′-GGTCAACAAATCATAAAGATATTGG-3′) with an amplicon size of 710 bp.

The PCR reaction was performed in 25 µL reaction volume, containing 12.5 µL of 2× PPP Master Mix (Top-Bio, Vestec, Czech Republic), 1 µL of each primer (10 µM), 9.5 µL of PCR-grade water, and 1 µL of DNA template. PCR conditions were as follows: initial denaturation at 94 °C for 1 min followed by 35 cycles of denaturation at 94 °C for 40 s, annealing at 44 °C for 40 s, and an extension at 72 °C for 1 min, with a final extension step at 72 °C for 10 min.

Another PCR was used to amplify a fragment of the mitochondrial 16S rDNA gene (~490 bp) of *Ixodes* spp. The primer pair Ixo-16S-F (5′-CTGCTCAATGATTWTTTAAATTGCTGTRG-3′) and Ixo-16S-R (5′-CCGGTCTGAACTCAGATCAWGT-3′) was designed manually based on the multiple alignment of the reference 16S rDNA sequences of *Ixodes* spp. retrieved from the GenBank database using the Primer3 v2.3.7 tool implemented in Geneious Prime v2025.1.3 (Biomatters Ltd., Auckland, New Zealand).

The PCR reaction mixture was prepared as described above. Amplification was performed under the following conditions: initial denaturation at 94 °C for 1 min followed by 35 cycles of denaturation at 94 °C for 15 s, annealing at 55 °C for 15 s, and extension at 72 °C for 45 s, with a final extension at 72 °C for 10 min. DNA extracted from an *I. ricinus* tick (unfed female) collected on vegetation and previously confirmed in our laboratory by morphological identification and molecular analysis was used as a positive control in both PCR assays. As negative controls, PCR-grade water (no-template negative control) and negative extraction control (sterile PBS) were used.

PCR products were visualized on 1.0% agarose gel with a Gel Red Nucleic Acid Stain (Biotinum, Fremont, CA, USA). Purification and Sanger sequencing was performed by Microsynth (Balgach, Switzerland). The resulting sequences were compared to reference sequences using BLASTn (https://blast.ncbi.nlm.nih.gov/Blast.cgi; accessed on 20 October 2025) and submitted to the GenBank database using the BankIt submission portal (https://www.ncbi.nlm.nih.gov/WebSub/; accessed on 20 October 2025).

### 2.4. Phylogenetic Analysis

Phylogenetic analyses were performed in MEGA v11 [24] using partial mitochondrial *COI* (642 bp) and 16S rDNA (465 bp) gene sequences of the *Ixodes* isolate obtained in this study, together with six *COI* and four 16S rDNA nucleotide sequences of *I. ariadnae* collected in Hungary, Belgium, Germany, Turkey, and Japan, and other *Ixodes* reference sequences retrieved from the GenBank database (accessed on 20 October 2025) (Supplementary Material, Tables S1 and S2). Multiple-sequence alignments were generated using the ClustalW algorithm (MEGA v11) (Supplementary Files S1 and S2). The best-fit nucleotide substitution models were selected based on the Bayesian Information Criterion (BIC) using the “Find Best DNA/Protein Models (ML)” option implemented in MEGA v11. Initial trees for the heuristic Maximum Likelihood (ML) search were generated automatically by applying Neighbor-Joining and BioNJ algorithms. Evolutionary history was inferred using the ML method under the Hasegawa–Kishino–Yano (HKY) nucleotide substitution model. For the *COI* dataset, rate heterogeneity among sites was modeled using a discrete Gamma distribution with five categories (+G) and a proportion of invariant sites (+I). For the 16S rDNA dataset, a discrete Gamma distribution with five categories (+G) was used. Node support was assessed by bootstrap analysis with 1000 replicates, and bootstrap values are shown next to the corresponding branches. Trees were drawn to scale, with branch lengths measured in the number of substitutions per site. The *COI* and 16S rDNA analyses resulted in final alignments of 593 and 381 nucleotide positions, respectively. Pairwise nucleotide sequence identity values (%) were calculated using Geneious Prime v2026.0.2 (Biomatters Ltd., Auckland, New Zealand) based on the final alignments. Genetic distances were calculated as uncorrected *p*-distances in MEGA v11 using the same datasets.

## 3. Results

### 3.1. Morphological Identification

**Figure 2:**
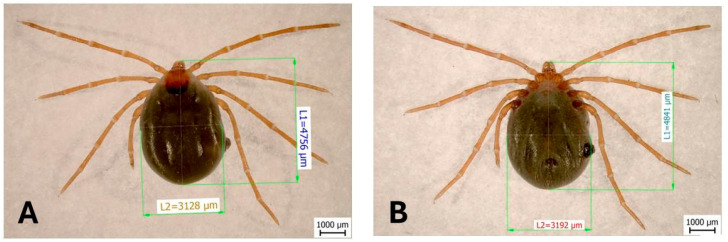
The dorsal view (**A**) of a collected female of *I. ariadnae*: L1—length of the body and L2—width of the body. The ventral view (**B**): L1—length of the body and L2—width of the body.

The total length of the tick’s body from the dorsal view was 4756 µm, and it was 4841 μm from the ventral view. The total width of the body from the dorsal view was 3128 µm, and it was 3192 μm from the ventral view. The scutum was rounded, slightly hexagonal, 1059 μm long, 942 μm wide, and had a shape index of 1.124. The idiosoma was sparsely covered with hairs on both dorsal and ventral sides (Figure 2).

**Figure 3:**
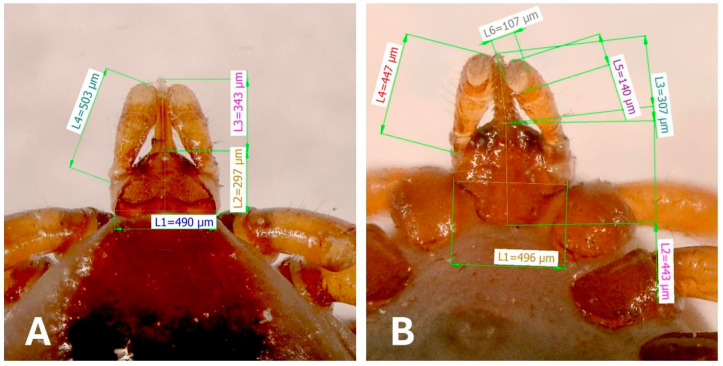
The dorsal view (**A**) of the gnathosoma of *I. ariadnae* with measurements of individual structures: L1—width of the basis capituli; L2—length of the basis capituli without the hypostome; L3—length of the hypostome; and L4—length of the palps. The ventral view (**B**): L1—width of the basis capituli; L2—length of the basis capituli without the hypostome; L3—length of the hypostome; L4—length of the palps; L5—width of the palp in the sagittal axis; and L6—width of the palp in the frontal axis.

The width of the basis capituli from the dorsal view was 490 µm, and 496 μm from the ventral view. The dorsal length of the palps was 503 μm and the dorsal width was 107 μm, with a visible junction of segments II and III. The hypostome dorsal length was 343 μm with prominent teeth. The palps from the ventral perspective were 447 µm long and 107 µm wide. The length of the hypostome on the ventral view was 307 µm (Figure 3).

Limb I (total length/tarsus) was 6393/1595 μm. Limb II (total length/tarsus) was 5473/1225 μm. Limb III (total length/tarsus) was 5823/1264 μm. Limb IV (total length/tarsus) was 5820/1263 μm. Haller’s organ oval was 283 x 82 μm. The coxae were convex and smooth. The genital pore was located between coxae III. The anal groove was anterior to the anus.

### 3.2. Sequence Identity and Phylogenetic Analysis

Both mitochondrial markers (*COI* and 16S rDNA) of the Slovak *I. ariadnae* isolate (designated as K265) were successfully amplified by conventional PCR and determined by Sanger sequencing. Partial *COI* (642 bp) and 16S rDNA (465 bp) sequences were deposited in the GenBank database under accession numbers PX474682 and PX474683, respectively.

The *COI* sequence (PX474682) exhibited 99.68–100% pairwise identity (Query coverage: 95–100%, E-value: 0.0) with *I. ariadnae* isolates from Hungary (KJ490306, LC769937), Germany (KR093169), and Turkey (ON527573). The 16S rDNA sequence (PX474683) showed 99.78–100% nucleotide identity (Query coverage: 85–100%, E-value: 0.0) with *I. ariadnae* isolates from Hungary (KM455969, KM455968), Germany (KR093170), and Turkey (ON540355). No other *Ixodes* species showed comparable levels of identity. For the *COI* fragment, the closest non-target species, *I. fujitai, I. vespertilionis,* and *I. canisuga*, showed only ~89% identity, and for the 16S rDNA sequence, *I. vespertilionis* and *I. canisuga* exhibited ~94% identity (Supplementary Material, Tables S3 and S4). These results provide molecular evidence confirming the taxonomic placement of the specimen within the *I. ariadnae* taxon.

**Figure 4:**
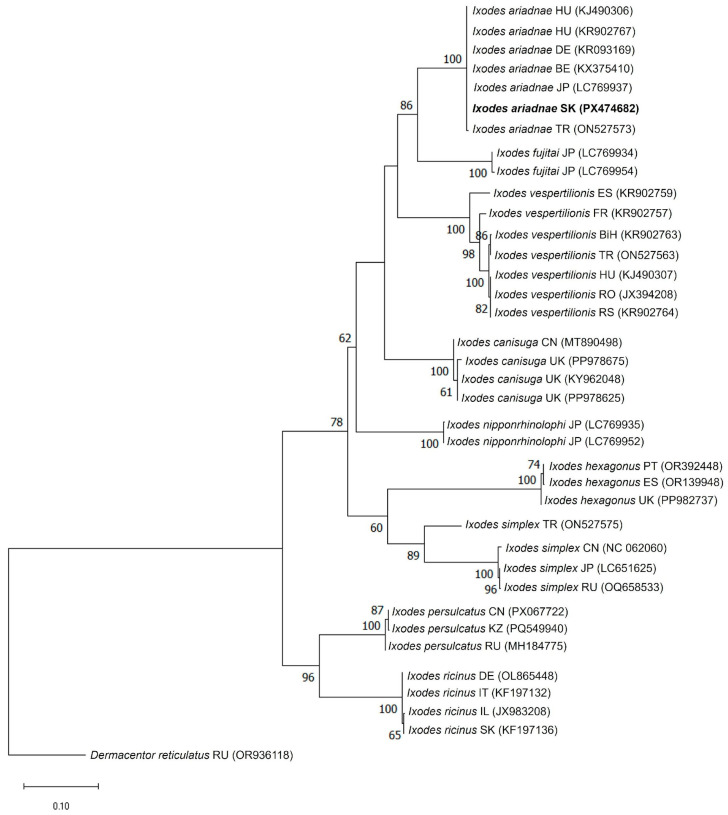
Phylogenetic analysis of *Ixodes ariadnae* isolate (K265) detected in this study. The phylogenetic tree was constructed based on the partial mitochondrial cytochrome c oxidase subunit I (*COI*) nucleotide sequences obtained in this study (PX474682) and reference strains of other *Ixodes* species available in the GenBank database. *Dermacentor reticulatus* (OR936118) was used as an out-group. The *COI* sequence of the *I. ariadnae* isolate (K265) identified in this study (PX474682) is indicated in bold. Bootstrap values ≥ 50% are depicted at their respective branches. Abbreviations: BE—Belgium, BiH—Bosnia and Herzegovina, CN—China, DE—Germany, ES—Spain, FR—France, HU—Hungary, IL—Israel, IT—Italy, JP—Japan, KZ—Kazakhstan, PT—Portugal, RO—Romania, RS—Serbia, RU—Russia, SK—Slovakia, TR—Turkey, and UK—United Kingdom.

**Figure 5:**
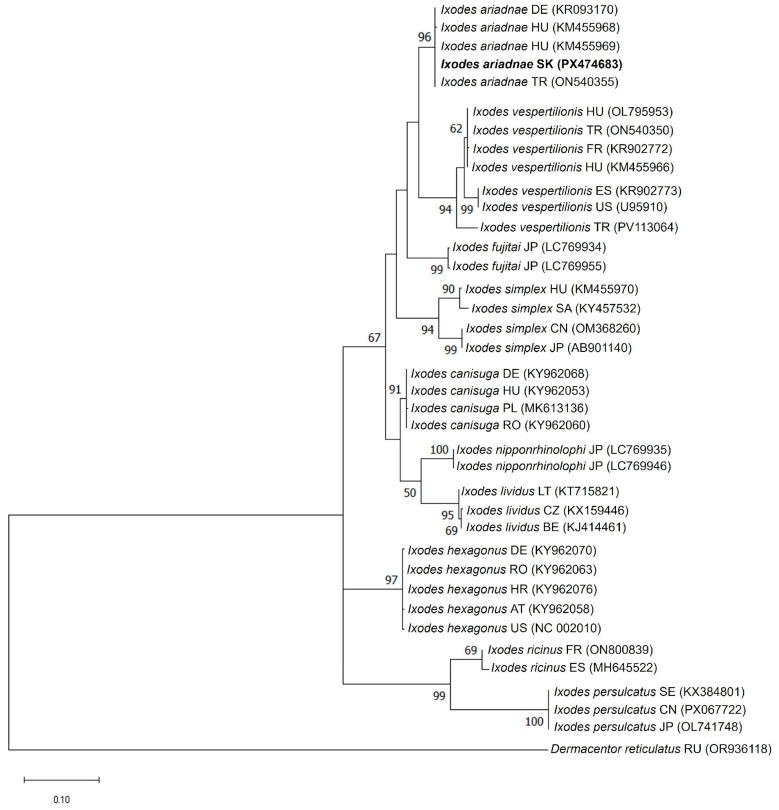
Phylogenetic analysis of *Ixodes ariadnae* isolate (K265) detected in this study. The phylogenetic tree was constructed based on the partial 16S rDNA nucleotide sequences obtained in this study (PX474683) and reference strains of other *Ixodes* species available in the GenBank database. *Dermacentor reticulatus* (OR936118) was used as an out-group. The 16S rDNA sequence of the *I. ariadnae* isolate (K265) identified in this study (PX474683) is indicated in bold. Bootstrap values ≥ 50% are depicted at their respective branches. Abbreviations: AT—Austria, BE—Belgium, CN—China, CZ—Czech Republic, DE—Germany, ES—Spain, FR—France, HR—Croatia, HU—Hungary, JP—Japan, LT—Lithuania, PL—Poland, RO—Romania, SA—Saudi Arabia, SK—Slovakia, TR—Turkey, UZ—Uzbekistan, and US—United States.

Phylogenetic analyses based on both mitochondrial markers consistently placed the newly identified Slovak specimen within the *I. ariadnae* clade (Figure 4 and Figure 5).

In the *COI*-based tree, the sequence from Slovakia (PX474682) clustered with *I. ariadnae* reference sequences from Europe (Hungary, Germany, Belgium, and Turkey) and Asia (Turkey and Japan), forming a well-supported monophyletic clade (100% bootstrap support). This clade was clearly separated from closely related species such as *I. vespertilionis* and *I. fujitai* (Figure 4). The 16S rDNA phylogeny exhibited a similar topology, with the Slovak *I. ariadnae* sequence (PX474683) clustering within the *I. ariadnae* clade together with sequences from Hungary, Germany, and Turkey (96% bootstrap support) (Figure 5).

Both analyses demonstrated clear genetic separation between *I. ariadnae* and other European bat-associated *Ixodes* species (*I. vespertilionis* and *I. simplex*). The consistent clustering patterns observed in both *COI* and 16S rDNA phylogenetic trees confirmed the identity of the analyzed specimen as *I. ariadnae* and provides molecular evidence for the first record of this tick species in Slovakia.

The nucleotide sequence comparison of the Slovak *I. ariadnae* isolate (K265) revealed 99.7–100% nucleotide identity (*p*-distances: 0.000–0.003) with *I. ariadnae* references in the *COI* gene and 99.7–100% (*p*-distances: 0.000–0.003) in the 16S rDNA gene (Supplementary Material, Figure S1). The sequence identity between the *I. ariadnae* K265 isolate and other *Ixodes* reference species ranged from 81.8–89.8% for the *COI* gene (*p*-distances: 0.102–0.182) and 83.1–93.5% for the 16S rDNA gene (*p*-distances: 0.065–0.169), highlighting the clear genetic distinctness of *I. ariadnae* from closely related taxa (Supplementary Material, Figure S2).

## 4. Discussion

In this paper, we describe the first detection of *Ixodes ariadnae* in Slovakia. To date, 25 hard ticks, including *I. ariadnae*, have been recorded in Slovakia [23,25,26,27,28,29,30,31,32].

Our findings show that although ixodid ticks are commonly found on vertebrate hosts in Slovakia [33,34], increased attention should be given to bats. Bats are an important part of the ecosystem. They provide ecosystem services [35], can serve as bioindicators of the presence of specific pollutants in the environment [36], and remain an interesting subject for research into new, as yet undescribed, host–parasite networks [8,37]. At the same time, because bats are capable of active flight, they play an important role in the transport of parasites and pathogens [38].

The importance of bat-associated parasitological studies is confirmed by the fact that this is the first record of *I. ariadnae* in Slovakia, although Hungarian locations adjacent to the Slovak border have been known since 2014 [16,39].

Our specimen of *I. ariadnae* was collected from a greater mouse-eared bat (*Myotis myotis*). The bat was found exhausted during the spring migration period (11 April 2025). As Slovak and Hungarian populations of *M. myotis* move between roosts in both countries [40], a plausible scenario is that the bat was returning from its Hungarian wintering grounds, where *I. ariadnae* is found. Local acquisition of the tick by the bat within Slovakia cannot be ruled out.

The collected tick specimen was first identified based on morphological characteristics. The individual morphological parameters of our specimen were comparable to, and correlated with, the biometric data of *I. ariadnae* from Hungary [16]. The length of the engorged female of *I. ariadnae* collected in a Hungarian cave was 6 mm, its legs were long (tarsus I 1.5 mm), its palps short (0.44 mm), with a hypostome length of 0.35 mm, and its scutum was oval and broad with a shape index of 1.2.

As *I. vespertilionis* and *I. simplex* also parasitize bats in Slovakia [41], several characteristics must be compared. The palps of *I. vespertilionis* are the longest and narrowest, whereas *I. ariadnae* and *I. simplex* have short, broad palps. *I. vespertilionis* has a sharp posterolateral flange on the basis capituli. The posterolateral flange in *I. ariadnae* is less produced. The two areae porosae of *I. vespertilionis* and *I. ariadnae* are circumscribed with densely situated small pits. The areae porosae of *I. simplex* are circumscribed by scattered pits. The scutum on the idiosoma is narrowest in *I. vespertilionis* and broadest in *I. ariadnae* [5].

The molecular data obtained in this study confirms the identity of the examined tick specimen as *I. ariadnae*. Both mitochondrial markers, *COI* and 16S rDNA, showed nearly complete sequence identity (99.8–100%) with *I. ariadnae* isolates previously reported from Central and Eastern Europe and Asia. The observed intraspecific genetic distances (*COI*: 0.000–0.003; 16S rDNA: 0.000–0.003) indicate extremely low genetic variability within the species, which is consistent with previous studies describing *I. ariadnae* as a genetically homogeneous and well-defined taxon [5,16,38].

The observed interspecific *p*-distances between *I. ariadnae* and other *Ixodes* species (*COI*: 0.14 ± 0.03; 16S rDNA: 0.09 ± 0.03; Supplementary Material, Table S5) are consistent with interspecific divergence values previously reported for *Ixodes* ticks (*COI*: 0.174 ± 0.052; 16S rDNA: 0.144 ± 0.059) [42]. This genetic separation between *I. ariadnae* and closely related taxa, such as *I. vespertilionis*, *I. canisuga*, and *I. fujitai*, confirms the molecular distinctness and evolutionary status of *I. ariadnae* within the *Ixodes* genus.

Phylogenetic analyses further supported the reliability of species-level identification. In both *COI*- and *16S*-based trees, the Slovak sequence clustered within the *I. ariadnae* clade with strong bootstrap support (100% and 96%, respectively). The monophyletic clustering with isolates from Europe (Hungary, Germany, and Belgium) and Asia (Turkey and Japan) suggests broad geographical consistency. These findings highlight the importance of integrating mitochondrial DNA markers with traditional morphological identification methods to ensure reliable and accurate species identification.

Based on this first record of *I. ariadnae*, it will be necessary to conduct targeted surveys of bat roosts and breeding colonies in Slovakia in the future. However, this type of study is limited by both the accessibility of bat roosts and the legislative framework. This is because legal permission must be obtained for targeted access to bat roosts, especially during the critical periods of rearing young and hibernation.

## 5. Conclusions

The detection of *I. ariadnae* in Slovakia expands the known distribution of this bat-associated tick species, which was previously confirmed in Europe (Hungary, Germany, Belgium, and Turkey) and Asia (Turkey and Japan). The present study provides the first molecular confirmation of *I. ariadnae* in Slovakia, contributing to a more complete understanding of the distribution and phylogeography of bat ticks in Europe. To examine whether *I. ariadnae* can be spread to Slovakia through bat migration, or whether it is currently hiddenly present in Slovakia, further detailed studies need to be carried out.

## Data Availability

All data presented in this article are available in the manuscript in tables and figures. The respective data can be found in GenBank at https://www.ncbi.nlm.nih.gov/genbank/ (accessed on 23 January 2026) under accession numbers PX474682 and PX474683. The specimen of *I. ariadnae* is placed in the depository of the authors.

## Supplementary Materials

The following supporting information can be downloaded at: https://www.mdpi.com/article/10.3390/ani16030391/s1, File S1. *COI* multiple sequence alignment used for phylogenetic analysis. File S2. 16S rDNA multiple sequence alignment used for phylogenetic analysis. Table S1. List of reference sequences used in this study for phylogenetic analysis based on the partial mitochondrial cytochrome c oxidase subunit I (*COI*) nucleotide sequences (accessed on 20 October 2025). Table S2. List of reference sequences used in this study for phylogenetic analysis based on the partial mitochondrial 16S rDNA nucleotide sequences (accessed on 20 October 2025). Table S3. List of sequences producing significant alignments with the partial *COI* sequence of *Ixodes ariadnae* isolate from this study with reference sequences deposited in GenBank database using the NCBI BLASTn algorithm (accessed 20 October 2025). Table S4. List of sequences producing significant alignments with the partial 16S rDNA sequence of *Ixodes ariadnae* isolate from this study with reference sequences deposited in GenBank database using the NCBI BLASTn algorithm (accessed 20 October 2025). Table S5. Interspecific *p*-distances between *Ixodes ariadnae* and other *Ixodes* species used in this study based on *COI* and 16S rDNA markers. Figure S1. Pairwise nucleotide sequence identity (%) of partial *COI* (A) and 16S rDNA (B) sequences of *Ixodes ariadnae* used in this study. Figure S2. Pairwise nucleotide sequence identity (%) of partial *COI* (A) and 16S rDNA (B) sequences of *Ixodes ariadnae* identified in this study (K265, indicated in bold) and other *Ixodes* reference sequences.
