# Supplementary material for: First Record of Ixodes ariadnae (Acari: Ixodidae) in Slovakia
Source: Animals (Basel). 2026 Jan 27;16(3):391. doi: 10.3390/ani16030391 (PMC12897055; doi:10.3390/ani16030391)
Supplement: Supplementary file 1 [file animals-16-00391-s001.zip › Supplementary Material.pdf]

Table S1. List of reference sequences used in this study for phylogenetic analysis based on the partial mitochondrial cytochrome c oxidase subunit I (*COI*) nucleotide sequences (accessed on 20 October 2025).

GenBank accession number	Species	Country
KR093169	*Ixodes ariadnae*	Germany
KX375410	*Ixodes ariadnae*	Belgium
KJ490306	*Ixodes ariadnae*	Hungary
KR902767	*Ixodes ariadnae*	Hungary
LC769937	*Ixodes ariadnae*	Japan
ON527573	*Ixodes ariadnae*	Turkey
MT890498	*Ixodes canisuga*	China
KY962048	*Ixodes canisuga*	United Kingdom
PP978625	*Ixodes canisuga*	United Kingdom
PP978675	*Ixodes canisuga*	United Kingdom
LC769934	*Ixodes fujitai*	Japan
LC769954	*Ixodes fujitai*	Japan
OR392448	*Ixodes hexagonus*	Portugal
OR139948	*Ixodes hexagonus*	Spain
PP982737	*Ixodes hexagonus*	United Kingdom
LC769935	*Ixodes nipponrhinolophi*	Japan
LC769952	*Ixodes nipponrhinolophi*	Japan
PX067722	*Ixodes persulcatus*	China
PQ549940	*Ixodes persulcatus*	Kazakhstan
MH184775	*Ixodes persulcatus*	Russia
OL865448	*Ixodes ricinus*	Germany
JX983208	*Ixodes ricinus*	Israel
KF197132	*Ixodes ricinus*	Italy
KF197136	*Ixodes ricinus*	Slovakia
NC_062060	*Ixodes simplex*	China
LC651625	*Ixodes simplex*	Japan
OQ658533	*Ixodes simplex*	Russia
ON527575	*Ixodes simplex*	Turkey
KR902763	*Ixodes vespertilionis*	Bosnia and Herzegovina
KR902757	*Ixodes vespertilionis*	France
KJ490307	*Ixodes vespertilionis*	Hungary
JX394208	*Ixodes vespertilionis*	Romania
KR902764	*Ixodes vespertilionis*	Serbia
KR902759	*Ixodes vespertilionis*	Spain
ON527563	*Ixodes vespertilionis*	Turkey
OR936118	*Dermacentor reticulatus*	Russia

Table S2. List of reference sequences used in this study for phylogenetic analysis based on the partial mitochondrial 16S rDNA nucleotide sequences (accessed on 20 October 2025).

GenBank accession number	Species	Country
KR093170	*Ixodes ariadnae*	Germany
KM455968	*Ixodes ariadnae*	Hungary
KM455969	*Ixodes ariadnae*	Hungary
ON540355	*Ixodes ariadnae*	Turkey
KY962068	*Ixodes canisuga*	Germany
KY962053	*Ixodes canisuga*	Hungary
MK613136	*Ixodes canisuga*	Poland
KY962060	*Ixodes canisuga*	Romania
LC769934	*Ixodes fujitai*	Japan
LC769955	*Ixodes fujitai*	Japan
KY962058	*Ixodes hexagonus*	Austria
KY962076	*Ixodes hexagonus*	Croatia
KY962070	*Ixodes hexagonus*	Germany
KY962063	*Ixodes hexagonus*	Romania
NC_002010	*Ixodes hexagonus*	USA
LC769935	*Ixodes nipponrhinolophi*	Japan
LC769946	*Ixodes nipponrhinolophi*	Japan
PX067722	*Ixodes persulcatus*	China
OL741748	*Ixodes persulcatus*	Japan
KX384801	*Ixodes persulcatus*	Sweden
ON800839	*Ixodes ricinus*	France
MH645522	*Ixodes ricinus*	Spain
KM455970	*Ixodes simplex*	Hungary
OM368260	*Ixodes simplex*	China
AB901140	*Ixodes simplex*	Japan
KY457532	*Ixodes simplex*	South Africa
KR902772	*Ixodes vespertilionis*	France
KM455966	*Ixodes vespertilionis*	Hungary
OL795953	*Ixodes vespertilionis*	Hungary
KR902773	*Ixodes vespertilionis*	Spain
ON540350	*Ixodes vespertilionis*	Turkey
PV113064	*Ixodes vespertilionis*	Turkey
U95910	*Ixodes vespertilionis*	USA
KX159446	*Ixodes lividus*	Czech Republic
KJ414461	*Ixodes lividus*	Belgium
KT715821	*Ixodes lividus*	Lithuania
OR936118	*Dermacentor reticulatus*	Russia

Table S3. List of sequences producing significant alignments with the partial *COI* sequence of *Ixodes ariadnae* isolate from this study with reference sequences deposited in GenBank database using the NCBI BLASTn algorithm (accessed 20 October 2025).

GenBank accession number	Species	Pairwise identity [%]	Query coverage [%]	E value
KJ490306.1	*Ixodes ariadnae*	100	100%	0.0
LC769937.1	*Ixodes ariadnae*	100	98%	0.0
KR093169.1	*Ixodes ariadnae*	100	95%	0.0
ON527573.1	*Ixodes ariadnae*	99.68	97%	0.0
LC769934.1	*Ixodes fujitai*	89.84	100%	0.0
LC769954.1	*Ixodes fujitai*	89.84	98%	0.0
PP978676.1	*Ixodes canisuga*	89.8	91%	0.0
LC036330.1	*Ixodes vespertilionis*	89.68	98%	0.0
MT890496.1	*Ixodes canisuga*	89.42	98%	0.0
MT890495.1	*Ixodes canisuga*	89.42	98%	0.0
MT890498.1	*Ixodes canisuga*	89.42	98%	0.0
MT890497.1	*Ixodes canisuga*	89.42	98%	0.0
LC797956.1	*Ixodes* sp.	89.38	100%	0.0
KY962030.1	*Ixodes canisuga*	89.26	98%	0.0
KY962048.1	*Ixodes canisuga*	89.26	98%	0.0
KY962049.1	*Ixodes canisuga*	89.26	98%	0.0
KY962047.1	*Ixodes canisuga*	89.26	98%	0.0
KY962017.1	*Ixodes canisuga*	89.26	98%	0.0
MT659136.1	*Ixodes canisuga*	89.26	98%	0.0
KY962045.1	*Ixodes canisuga*	89.26	98%	0.0
PP503327.1	*Ixodes* sp.	89.22	98%	0.0
PP048274.1	*Ixodes canisuga*	89.16	95%	0.0
PP978675.1	*Ixodes canisuga*	89.15	99%	0.0
MT659130.1	*Ixodes canisuga*	89.1	98%	0.0
KY962023.1	*Ixodes canisuga*	89.1	98%	0.0
KY962022.1	*Ixodes canisuga*	89.1	98%	0.0
KY962051.1	*Ixodes canisuga*	89.1	98%	0.0
MT659137.1	*Ixodes canisuga*	89.1	98%	0.0
KY962037.1	*Ixodes canisuga*	89.1	98%	0.0
KY962038.1	*Ixodes canisuga*	89.1	98%	0.0
PX239320.1	*Ixodes crenulatus*	89.08	98%	0.0
PX239325.1	*Ixodes crenulatus*	89.08	98%	0.0
PP503326.1	*Ixodes* sp.	89.06	98%	0.0
OM368263.1	*Ixodes vespertilionis*	89.06	100%	0.0
PP978625.1	*Ixodes canisuga*	88.99	99%	0.0
PP978650.1	*Ixodes canisuga*	88.99	99%	0.0
PP978621.1	*Ixodes canisuga*	88.99	99%	0.0
PP978672.1	*Ixodes canisuga*	88.99	99%	0.0
PV061061.1	*Ixodes canisuga*	88.98	99%	0.0
KY962050.1	*Ixodes canisuga*	88.94	98%	0.0
KY962013.1	*Ixodes canisuga*	88.94	98%	0.0
KY962031.1	*Ixodes canisuga*	88.94	98%	0.0

KY962044.1	*Ixodes canisuga*	88.94	98%	0.0
KY962021.1	*Ixodes canisuga*	88.94	98%	0.0
KY962016.1	*Ixodes canisuga*	88.94	98%	0.0
KY962040.1	*Ixodes canisuga*	88.94	98%	0.0
PX239319.1	*Ixodes crenulatus*	88.92	98%	0.0
PP048133.1	*Ixodes canisuga*	88.92	98%	0.0
PX239326.1	*Ixodes crenulatus*	88.92	98%	0.0
PP047756.1	*Ixodes canisuga*	88.92	98%	0.0
PP047788.1	*Ixodes canisuga*	88.92	98%	0.0
PP079465.1	*Ixodes lanigeri*	88.89	98%	0.0
PP978661.1	*Ixodes canisuga*	88.84	99%	0.0
KY962025.1	*Ixodes canisuga*	88.78	98%	0.0
KY962039.1	*Ixodes canisuga*	88.78	98%	0.0
KY962012.1	*Ixodes canisuga*	88.78	98%	0.0
PP047818.1	*Ixodes canisuga*	88.77	98%	0.0
PX239318.1	*Ixodes crenulatus*	88.77	98%	0.0
PP048138.1	*Ixodes canisuga*	88.77	98%	0.0
KX218106.1	*Ixodes canisuga*	88.73	99%	0.0
ON527562.1	*Ixodes vespertilionis*	88.53	92%	0.0
ON527569.1	*Ixodes vespertilionis*	88.53	92%	0.0
PX672559.1	*Ixodes vespertilionis*	88.46	93%	0.0
PX442027.1	*Ixodes crenulatus*	88.46	100%	0.0
NC_085484.1	*Ixodes crenulatus*	88.46	100%	0.0
ON527566.1	*Ixodes vespertilionis*	88.36	92%	0.0
ON527561.1	*Ixodes vespertilionis*	88.36	92%	0.0
ON527567.1	*Ixodes vespertilionis*	88.36	92%	0.0
ON527568.1	*Ixodes vespertilionis*	88.36	92%	0.0
ON527563.1	*Ixodes vespertilionis*	88.36	92%	0.0
NC_058244.1	*Ixodes vespertilionis*	88.3	100%	0.0
PX672563.1	*Ixodes vespertilionis*	88.29	93%	0.0
KR902758.1	*Ixodes vespertilionis*	88.19	99%	0.0
KR902757.1	*Ixodes vespertilionis*	87.87	99%	0.0
KJ490307.1	*Ixodes vespertilionis*	87.73	100%	0.0
KJ490308.1	*Ixodes vespertilionis*	87.73	100%	0.0
KR902764.1	*Ixodes vespertilionis*	87.72	99%	0.0
KR902759.1	*Ixodes vespertilionis*	87.72	99%	0.0
KR902761.1	*Ixodes vespertilionis*	87.56	99%	0.0
LC769936.1	*Ixodes vespertilionis*	87.54	98%	0.0
KR902766.1	*Ixodes vespertilionis*	87.54	98%	0.0
KJ490311.1	*Ixodes vespertilionis*	87.54	100%	0.0
KJ490309.1	*Ixodes vespertilionis*	87.42	100%	0.0
KR902763.1	*Ixodes vespertilionis*	87.4	99%	0.0
KR902762.1	*Ixodes vespertilionis*	87.4	99%	0.0
JX394205.1	*Ixodes vespertilionis*	87.38	98%	0.0
JX394207.1	*Ixodes vespertilionis*	87.38	98%	0.0
JX394208.1	*Ixodes vespertilionis*	87.38	98%	0.0
LC067571.1	*Ixodes vespertilionis*	87.3	98%	0.0
LC067573.1	*Ixodes vespertilionis*	87.3	98%	0.0
KJ490310.1	*Ixodes vespertilionis*	87.27	100%	0.0
KR902760.1	*Ixodes vespertilionis*	87.24	99%	0.0

JX394206.1	*Ixodes vespertilionis*	87.22	98%	0.0
MK450319.1	*Ixodes collaris*	87.2	99%	0.0
MK450318.1	*Ixodes collaris*	87.2	99%	0.0
KR902756.1	*Ixodes collaris*	87.2	99%	0.0
AB231667.1	*Ixodes vespertilionis*	87.19	100%	0.0
LC769935.1	*Ixodes nipponrhinolophi*	87.19	100%	0.0
LC067572.1	*Ixodes vespertilionis*	87.14	98%	0.0

Table S4. List of sequences producing significant alignments with the partial 16S rDNA sequence of *Ixodes ariadnae* isolate from this study with reference sequences deposited in GenBank database using the NCBI BLASTn algorithm (accessed 20 October 2025).

GenBank accession number	Species	Pairwise identity [%]	Query coverage [%]	E value
KM455969.1	*Ixodes ariadnae*	100	100%	0.0
ON540355.1	*Ixodes ariadnae*	100	89%	0.0
KR093170.1	*Ixodes ariadnae*	100	85%	0.0
KM455968.1	*Ixodes ariadnae*	99.78	100%	0.0
PP505540.1	*Ixodes* sp.	94.87	88%	1.00E-177
KM455966.1	*Ixodes vespertilionis*	94.84	100%	0.0
KM455967.1	*Ixodes vespertilionis*	94.84	100%	0.0
MK613136.1	*Ixodes canisuga*	94.83	96%	0.0
MK613137.1	*Ixodes canisuga*	94.83	96%	0.0
KY962053.1	*Ixodes canisuga*	94.81	87%	2.00E-175
KY962068.1	*Ixodes canisuga*	94.81	87%	2.00E-175
KY962072.1	*Ixodes canisuga*	94.81	87%	2.00E-175
KY962065.1	*Ixodes canisuga*	94.81	87%	2.00E-175
KY962055.1	*Ixodes canisuga*	94.81	87%	2.00E-175
KY962075.1	*Ixodes canisuga*	94.81	87%	2.00E-175
KY962060.1	*Ixodes canisuga*	94.81	87%	2.00E-175
OM200059.1	*Ixodes canisuga*	94.75	86%	1.00E-172
OP902286.1	*Ixodes canisuga*	94.65	88%	1.00E-177
MT889694.1	*Ixodes canisuga*	94.65	88%	1.00E-177
MT889698.1	*Ixodes canisuga*	94.65	88%	1.00E-177
MT889696.1	*Ixodes canisuga*	94.65	88%	1.00E-177
MT889697.1	*Ixodes canisuga*	94.65	88%	1.00E-177
MT889695.1	*Ixodes canisuga*	94.65	88%	1.00E-177
OP902283.1	*Ixodes canisuga*	94.65	88%	1.00E-177
PP505539.1	*Ixodes* sp.	94.62	88%	5.00E-176
MT658764.1	*Ixodes canisuga*	94.58	87%	3.00E-174
KY962061.1	*Ixodes canisuga*	94.57	87%	9.00E-174
KY962069.1	*Ixodes canisuga*	94.57	87%	9.00E-174
KY962056.1	*Ixodes canisuga*	94.57	87%	9.00E-174
MT658762.1	*Ixodes canisuga*	94.57	87%	9.00E-174
KY962071.1	*Ixodes canisuga*	94.57	87%	9.00E-174
KY962064.1	*Ixodes canisuga*	94.57	87%	9.00E-174
KY962073.1	*Ixodes canisuga*	94.57	87%	9.00E-174
KY962074.1	*Ixodes canisuga*	94.57	87%	9.00E-174
PP081435.1	*Ixodes* sp.	94.55	90%	0.0
OL795953.1	*Ixodes vespertilionis*	94.48	97%	0.0
LC797956.1	*Ixodes* sp.	94.42	100%	0.0
PV113064.1	*Ixodes vespertilionis*	94.41	100%	0.0
OL795934.1	*Ixodes vespertilionis*	94.26	97%	0.0
ON540350.1	*Ixodes vespertilionis*	94.22	89%	5.00E-176
ON540349.1	*Ixodes vespertilionis*	94.22	89%	5.00E-176
OM368263.1	*Ixodes vespertilionis*	94.21	100%	0.0

NC_085484.1	*Ixodes crenulatus*	94.19	100%	0.0
MT261040.1	*Ixodes lividus*	94.14	99%	0.0
KX218233.1	*Ixodes canisuga*	94.13	88%	4.00E-172
PQ740499.1	*Ixodes vespertilionis*	94.1	88%	4.00E-172
ON540352.1	*Ixodes vespertilionis*	93.98	89%	3.00E-174
ON540351.1	*Ixodes vespertilionis*	93.98	89%	3.00E-174
U95910.1	*Ixodes vespertilionis*	93.75	100%	0.0
ON540353.1	*Ixodes vespertilionis*	93.73	89%	1.00E-172
ON540354.1	*Ixodes vespertilionis*	93.73	89%	1.00E-172
KJ414461.1	*Ixodes lividus*	93.49	99%	0.0
LC769934.1	*Ixodes fujitai*	93.33	100%	0.0
LC036329.1	*Ixodes vespertilionis*	93.24	89%	3.00E-169
NC_058244.1	*Ixodes vespertilionis*	93.12	100%	0.0
OL795943.1	*Ixodes simplex*	92.79	97%	0.0
OL795961.1	*Ixodes simplex*	92.79	97%	0.0
MG651937.1	*Ixodes canisuga*	92.65	97%	7.00E-180
OL795960.1	*Ixodes simplex*	92.58	97%	0.0
OL795945.1	*Ixodes simplex*	92.58	97%	0.0
OL795949.1	*Ixodes simplex*	92.58	97%	0.0
OL795954.1	*Ixodes simplex*	92.58	97%	0.0
OL795940.1	*Ixodes simplex*	92.58	97%	0.0
OL795962.1	*Ixodes simplex*	92.58	97%	0.0
OL795937.1	*Ixodes simplex*	92.58	97%	0.0
OL795959.1	*Ixodes simplex*	92.58	97%	0.0
OL795944.1	*Ixodes simplex*	92.58	97%	0.0
OL795956.1	*Ixodes simplex*	92.58	97%	0.0
OL795955.1	*Ixodes simplex*	92.58	97%	0.0
OL795946.1	*Ixodes simplex*	92.58	97%	0.0
OL795939.1	*Ixodes simplex*	92.58	97%	0.0
OL795963.1	*Ixodes simplex*	92.58	97%	0.0
OL795941.1	*Ixodes simplex*	92.58	97%	0.0
OL795931.1	*Ixodes simplex*	92.58	97%	0.0
OL795938.1	*Ixodes simplex*	92.58	97%	0.0
OL795936.1	*Ixodes simplex*	92.58	97%	0.0
OL795952.1	*Ixodes simplex*	92.58	97%	0.0
OL795958.1	*Ixodes simplex*	92.58	97%	0.0
OL795947.1	*Ixodes simplex*	92.58	97%	0.0
OL795948.1	*Ixodes simplex*	92.58	97%	0.0
OL795951.1	*Ixodes simplex*	92.58	97%	0.0
OL795957.1	*Ixodes simplex*	92.58	97%	0.0
OL795950.1	*Ixodes simplex*	92.58	97%	0.0
OL795942.1	*Ixodes simplex*	92.58	97%	0.0
LC769935.1	*Ixodes nipponrhinolophi*	92.52	100%	0.0
LC769933.1	*Ixodes fuliginosus*	92.52	100%	0.0
OR197646.1	*Ixodes vespertilionis*	92.52	100%	0.0
KY457532.1	*Ixodes simplex*	92.51	100%	0.0
KY457531.1	*Ixodes simplex*	92.51	100%	0.0
KM455970.1	*Ixodes simplex*	92.39	100%	0.0
OL795935.1	*Ixodes simplex*	92.36	97%	7.00E-180
OL795929.1	*Ixodes simplex*	92.36	97%	7.00E-180

OL795932.1	*Ixodes simplex*	92.36	97%	7.00E-180
OL795933.1	*Ixodes simplex*	92.36	97%	7.00E-180
OL795930.1	*Ixodes simplex*	92.36	97%	7.00E-180
MW132810.1	*Ixodes simplex*	92.13	95%	3.00E-173
NC_062060.1	*Ixodes simplex*	92.11	100%	0.0
KJ414453.1	*Ixodes arboricola*	91.56	99%	2.00E-176
AF113928.1	*Ixodes auritulus*	90.56	100%	9.00E-169

Table S5. Interspecific *p*-distances between *Ixodes ariadnae* and other *Ixodes* species used in this study based on *COI* and 16S rDNA markers.

**Group comparison**	***COI*** ***p*-distance**	**16S rDNA** ***p*-distance**
Ixodes ariadnae – I. canisuga	0.113	0.053
Ixodes ariadnae – Ixodes fujitai	0.103	0.068
Ixodes ariadnae – Ixodes hexagonus	0.180	0.112
Ixodes ariadnae – Ixodes nipponrhinolophi	0.132	0.081
Ixodes ariadnae – Ixodes persulcatus	0.177	0.146
Ixodes ariadnae – Ixodes ricinus	0.159	0.137
Ixodes ariadnae – Ixodes simplex	0.149	0.082
Ixodes ariadnae – Ixodes vespertilionis	0.127	0.061
Ixodes ariadnae – Ixodes lividus	-	0.070

Figure S1. Pairwise nucleotide sequence identity (%) of partial *COI* (A) and 16S rDNA (B) sequences of *Ixodes ariadnae* used in this study. Isolate identified in this study (K265) is indicated in bold.

A

	Ixodes ariadnae Belgium (KX375410)	*Ixodes ariadnae* Germany (KR093169)	*Ixodes ariadnae* Hungary (KJ490306)	*Ixodes ariadnae* Hungary (KR902767)	*Ixodes ariadnae* Japan (LC769937)	***Ixodes ariadnae* Slovakia (PX474682)**	*Ixodes ariadnae* Turkey (ON527573)
Ixodes ariadnae Belgium (KX375410)	ID	100	100	100	100	100	99.7
Ixodes ariadnae Germany (KR093169)	100	ID	100	100	100	100	99.7
Ixodes ariadnae Hungary (KJ490306)	100	100	ID	100	100	100	99.7
Ixodes ariadnae Hungary (KR902767)	100	100	100	ID	100	100	99.7
Ixodes ariadnae Japan (LC769937)	100	100	100	100	ID	100	99.7
***Ixodes ariadnae* Slovakia (PX474682)**	100	100	100	100	100	ID	99.7
Ixodes ariadnae Turkey (ON527573)	99.7	99.7	99.7	99.7	99.7	99.7	ID

B

	Ixodes ariadnae Germany (KR093170)	*Ixodes ariadnae* Hungary (KM455968)	*Ixodes ariadnae* Hungary (KM455969)	***Ixodes ariadnae* Slovakia (PX474683)**	*Ixodes ariadnae* Turkey (ON540355)
Ixodes ariadnae Germany (KR093170)	ID	99.7	100	100	100
Ixodes ariadnae Hungary (KM455968)	99.7	ID	99.7	99.7	99.7
Ixodes ariadnae Hungary (KM455969)	100	99.7	ID	100	100
***Ixodes ariadnae* Slovakia (PX474683)**	100	99.7	100	ID	100
Ixodes ariadnae Turkey (ON540355)	100	99.7	100	100	ID

Figure S2. Pairwise nucleotide sequence identity (%) of partial *COI* (A) and 16S rDNA (B) sequences of *Ixodes ariadnae* identified in this study (K265, indicated in bold) and other *Ixodes* reference sequences.

A

	Ixodes canisuga China (MT890498)	*Ixodes canisuga* UK (KY962048)	*Ixodes canisuga* UK (PP978625)	*Ixodes canisuga* UK (PP978675)	*Ixodes fujitai* Japan (LC769934)	*Ixodes fujitai* Japan (LC769954)	*Ixodes hexagonus* Portugal (OR392448)	*Ixodes hexagonus* Spain (OR139948)	*Ixodes hexagonus* United Kingdom (PP982737)	*Ixodes nipponrhinolophi* Japan (LC769935)	*Ixodes nipponrhinolophi* Japan (LC769952)	*Ixodes persulcatus* China (PX067722)	*Ixodes persulcatus* Kazachstan (PQ549940)	*Ixodes persulcatus* Rusia (MH184775)	*Ixodes ricinus* Germany (OL865448)	*Ixodes ricinus* Israel (JX983208)	*Ixodes ricinus* Italy (KF197132)	*Ixodes ricinus* Slovakia (KF197136)	*Ixodes simplex* China (NC 062060)	*Ixodes simplex* Japan (LC651625)	*Ixodes simplex* Russia (OQ658533)	*Ixodes simplex* Turkey (ON527575)	*Ixodes vespertilionis* Bosnia and Herzegovina (KR902763)	*Ixodes vespertilionis* France (KR902757)	*Ixodes vespertilionis* Hungary (KJ490307)	*Ixodes vespertilionis* Romania (JX394208)	*Ixodes vespertilionis* Serbia (KR902764)	*Ixodes vespertilionis* Spain (KR902759)	*Ixodes vespertilionis* Turkey (ON527563)	***Ixodes ariadnae* Slovakia (PX474682)**
Ixodes canisuga China (MT890498)	ID	99.5	99.5	99	88.4	88	85.3	85.3	85.3	86.2	85.8	85.2	85	85.3	87	86.7	87	86.8	85.8	85.8	85.7	87.5	87.4	87.4	87.5	87.5	87.5	87	87.5	88.9
Ixodes canisuga UK (KY962048)	99.5	ID	100	99.5	88.5	88.2	84.8	84.8	84.8	86	85.7	85.3	85.2	85.5	86.8	86.8	86.8	86.7	85.3	85.7	85.5	87.2	87	87	87.2	87.2	87.2	86.7	87.2	88.7
Ixodes canisuga UK (PP978625)	99.5	100	ID	99.5	88.5	88.2	84.8	84.8	84.8	86	85.7	85.3	85.2	85.5	86.8	86.8	86.8	86.7	85.3	85.7	85.5	87.2	87	87	87.2	87.2	87.2	86.7	87.2	88.7
Ixodes canisuga UK (PP978675)	99	99.5	99.5	ID	88.5	88.2	85	85	85	85.7	85.3	85	84.8	85.2	86.8	86.8	86.8	86.7	85.5	85.8	85.7	87.2	87	87	87.2	87.2	87.2	86.7	87.2	88.9
Ixodes fujitai Japan (LC769934)	88.4	88.5	88.5	88.5	ID	99.7	83	83	83.1	84.7	84.5	84.1	84	84.3	84.1	84.1	84.1	84	82.8	83.1	83	85.3	86	86.3	86.2	86.2	86.2	85.7	86.2	89.7
Ixodes fujitai Japan (LC769954)	88	88.2	88.2	88.2	99.7	ID	82.6	82.6	82.8	84.7	84.5	84	83.8	84.1	84	84	84	83.8	82.6	83	82.8	85	85.8	86.2	86	86	86	85.3	86	89.9
Ixodes hexagonus Portugal (OR392448)	85.3	84.8	84.8	85	83	82.6	ID	99.8	99.7	80.8	80.9	82.8	82.6	83.1	80.6	80.3	80.6	80.4	84.3	84.5	84.3	84.5	82.6	83.5	82.6	82.6	82.6	82.5	82.6	82
Ixodes hexagonus Spain (OR139948)	85.3	84.8	84.8	85	83	82.6	99.8	ID	99.5	80.6	80.8	83	82.8	83.3	80.6	80.3	80.6	80.4	84.5	84.7	84.5	84.7	82.6	83.5	82.6	82.6	82.6	82.5	82.6	82
Ixodes hexagonus United Kingdom (PP982737)	85.3	84.8	84.8	85	83.1	82.8	99.7	99.5	ID	80.9	81.1	82.8	82.6	83.1	80.8	80.4	80.8	80.6	84.5	84.7	84.5	84.7	82.6	83.5	82.6	82.6	82.6	82.5	82.6	82.3
Ixodes nipponrhinolophi Japan (LC769935)	86.2	86	86	85.7	84.7	84.7	80.8	80.6	80.9	ID	99.7	85.2	85	85.3	84.3	84	84.3	84.1	84.3	84.7	84.5	86	85.5	86.2	86	86	86	86.2	85.7	86.8
Ixodes nipponrhinolophi Japan (LC769952)	85.8	85.7	85.7	85.3	84.5	84.5	80.9	80.8	81.1	99.7	ID	85.2	85	85.3	84	83.6	84	83.8	84.1	84.5	84.3	86	85.2	86.2	85.7	85.7	85.7	86.2	85.3	86.8
Ixodes persulcatus China (PX067722)	85.2	85.3	85.3	85	84.1	84	82.8	83	82.8	85.2	85.2	ID	99.8	99.7	88.9	88.9	88.9	88.7	82.8	83.1	83	83.1	80.8	81.8	81.1	81.1	81.1	81.3	80.9	82.3
Ixodes persulcatus Kazachstan (PQ549940)	85	85.2	85.2	84.8	84	83.8	82.6	82.8	82.6	85	85	99.8	ID	99.5	88.7	88.7	88.7	88.5	83	83.3	83.1	83	80.6	81.6	80.9	80.9	80.9	81.1	80.8	82.1
Ixodes persulcatus Rusia (MH184775)	85.3	85.5	85.5	85.2	84.3	84.1	83.1	83.3	83.1	85.3	85.3	99.7	99.5	ID	89	89	89	88.9	83	83.3	83.1	83.3	80.9	82	81.3	81.3	81.3	81.5	81.1	82.5
Ixodes ricinus Germany (OL865448)	87	86.8	86.8	86.8	84.1	84	80.6	80.6	80.8	84.3	84	88.9	88.7	89	ID	99.7	100	99.8	82.5	82.6	82.5	82.6	82.5	82.5	82.6	82.6	82.6	82.8	82.6	84.3
Ixodes ricinus Israel (JX983208)	86.7	86.8	86.8	86.8	84.1	84	80.3	80.3	80.4	84	83.6	88.9	88.7	89	99.7	ID	99.7	99.8	82.5	82.6	82.5	82.3	82.1	82.1	82.3	82.3	82.3	82.5	82.3	84
Ixodes ricinus Italy (KF197132)	87	86.8	86.8	86.8	84.1	84	80.6	80.6	80.8	84.3	84	88.9	88.7	89	100	99.7	ID	99.8	82.5	82.6	82.5	82.6	82.5	82.5	82.6	82.6	82.6	82.8	82.6	84.3
Ixodes ricinus Slovakia (KF197136)	86.8	86.7	86.7	86.7	84	83.8	80.4	80.4	80.6	84.1	83.8	88.7	88.5	88.9	99.8	99.8	99.8	ID	82.6	82.8	82.6	82.5	82.3	82.3	82.5	82.5	82.5	82.6	82.5	84.1
Ixodes simplex China (NC 062060)	85.8	85.3	85.3	85.5	82.8	82.6	84.3	84.5	84.5	84.3	84.1	82.8	83	83	82.5	82.5	82.5	82.6	ID	99.3	99.2	89.7	82.6	82.5	83	83	83	82	82.8	84.8
Ixodes simplex Japan (LC651625)	85.8	85.7	85.7	85.8	83.1	83	84.5	84.7	84.7	84.7	84.5	83.1	83.3	83.3	82.6	82.6	82.6	82.8	99.3	ID	99.8	89.9	82.8	82.6	83.1	83.1	83.1	82.1	83	85.2
Ixodes simplex Russia (OQ658533)	85.7	85.5	85.5	85.7	83	82.8	84.3	84.5	84.5	84.5	84.3	83	83.1	83.1	82.5	82.5	82.5	82.6	99.2	99.8	ID	89.7	82.6	82.5	83	83	83	82	82.8	85
Ixodes simplex Turkey (ON527575)	87.5	87.2	87.2	87.2	85.3	85	84.5	84.7	84.7	86	86	83.1	83	83.3	82.6	82.3	82.6	82.5	89.7	89.9	89.7	ID	84.3	84.7	84.8	84.8	84.8	84.5	84.5	85.7
Ixodes vespertilionis Bosnia and Herzegovina (KR902763)	87.4	87	87	87	86	85.8	82.6	82.6	82.6	85.5	85.2	80.8	80.6	80.9	82.5	82.1	82.5	82.3	82.6	82.8	82.6	84.3	ID	97.6	99.5	99.5	99.5	95.1	99.8	87.2
Ixodes vespertilionis France (KR902757)	87.4	87	87	87	86.3	86.2	83.5	83.5	83.5	86.2	86.2	81.8	81.6	82	82.5	82.1	82.5	82.3	82.5	82.6	82.5	84.7	97.6	ID	97.8	97.8	97.8	95.6	97.8	87.7
Ixodes vespertilionis Hungary (KJ490307)	87.5	87.2	87.2	87.2	86.2	86	82.6	82.6	82.6	86	85.7	81.1	80.9	81.3	82.6	82.3	82.6	82.5	83	83.1	83	84.8	99.5	97.8	ID	100	100	95.3	99.7	87.4
Ixodes vespertilionis Romania (JX394208)	87.5	87.2	87.2	87.2	86.2	86	82.6	82.6	82.6	86	85.7	81.1	80.9	81.3	82.6	82.3	82.6	82.5	83	83.1	83	84.8	99.5	97.8	100	ID	100	95.3	99.7	87.4
Ixodes vespertilionis Serbia (KR902764)	87.5	87.2	87.2	87.2	86.2	86	82.6	82.6	82.6	86	85.7	81.1	80.9	81.3	82.6	82.3	82.6	82.5	83	83.1	83	84.8	99.5	97.8	100	100	ID	95.3	99.7	87.4
Ixodes vespertilionis Spain (KR902759)	87	86.7	86.7	86.7	85.7	85.3	82.5	82.5	82.5	86.2	86.2	81.3	81.1	81.5	82.8	82.5	82.8	82.6	82	82.1	82	84.5	95.1	95.6	95.3	95.3	95.3	ID	95.3	87.2
Ixodes vespertilionis Turkey (ON527563)	87.5	87.2	87.2	87.2	86.2	86	82.6	82.6	82.6	85.7	85.3	80.9	80.8	81.1	82.6	82.3	82.6	82.5	82.8	83	82.8	84.5	99.8	97.8	99.7	99.7	99.7	95.3	ID	87.4
***Ixodes ariadnae* Slovakia (PX474682)**	88.9	88.7	88.7	88.9	89.7	89.9	82	82	82.3	86.8	86.8	82.3	82.1	82.5	84.3	84	84.3	84.1	84.8	85.2	85	85.7	87.2	87.7	87.4	87.4	87.4	87.2	87.4	ID

80 85 90 95 100

B

	Ixodes ricinus France (ON800839)	*Ixodes ricinus* Spain (MH645522)	*Ixodes canisuga* Germany (KY962068)	*Ixodes canisuga* Hungary (KY962053)	*Ixodes canisuga* Poland (MK613136)	*Ixodes canisuga* Romania (KY962060)	*Ixodes fujitai* Japan (LC769934)	*Ixodes fujitai* Japan (LC769955)	*Ixodes hexagonus* USA (NC 002010)	*Ixodes hexagonus* Austria (KY962058)	*Ixodes hexagonus* Croatia (KY962076)	*Ixodes hexagonus* Germany (KY962070)	*Ixodes hexagonus* Romania (KY962063)	*Ixodes lividus* Belgium (KJ414461)	*Ixodes lividus* Czech Republic (KX159446)	*Ixodes lividus* Lithuania (KT715821)	*Ixodes nipponrhinolophi* Japan (LC769935)	*Ixodes nipponrhinolophi* Japan (LC769946)	*Ixodes persulcatus* China (PX067722)	*Ixodes persulcatus* Japan (OL741748)	*Ixodes persulcatus* Sweden (KX384801)	*Ixodes simplex* China (OM368260)	*Ixodes simplex* Hungary (KM455970)	*Ixodes simplex* Japan (AB901140)	*Ixodes simplex* South Africa (KY457532)	*Ixodes vespertilionis* France (KR902772)	*Ixodes vespertilionis* Hungary (KM455966)	*Ixodes vespertilionis* Hungary (OL795953)	*Ixodes vespertilionis* Spain (KR902773)	*Ixodes vespertilionis* Turkey (ON540350)	*Ixodes vespertilionis* Turkey (PV113064)	*Ixodes vespertilionis* USA (U95910)	***Ixodes ariadnae* Slovakia (PX474683)**
Ixodes ricinus France (ON800839)	ID	98.9	86.8	86.8	86.5	86.8	84.1	83.6	85.9	85.9	86.2	85.9	86.2	84.4	84.9	84.4	85.1	85.4	87.6	87.6	87.6	86.1	86	86.3	86.3	85.4	85.1	85.1	83.8	85.1	83.8	83.8	85.5
Ixodes ricinus Spain (MH645522)	98.9	ID	87.3	87.3	87	87.3	83.9	83.3	85.9	85.9	86.2	85.9	86.2	84.4	84.9	84.4	85.4	85.6	86.8	86.8	86.8	85.5	85.5	85.8	85.7	85.4	85.1	85.1	83.8	85.1	83.8	83.8	84.9
Ixodes canisuga Germany (KY962068)	86.8	87.3	ID	100	99.7	100	92.2	92.2	85.1	85.1	85.3	85.1	85.3	94.1	94	94.3	92	92	84.4	84.4	84.4	90.6	90.6	90.9	91.6	95.1	94.9	94.9	93.8	94.9	93	93.8	93.5
Ixodes canisuga Hungary (KY962053)	86.8	87.3	100	ID	99.7	100	92.2	92.2	85.1	85.1	85.3	85.1	85.3	94.1	94	94.3	92	92	84.4	84.4	84.4	90.6	90.6	90.9	91.6	95.1	94.9	94.9	93.8	94.9	93	93.8	93.5
Ixodes canisuga Poland (MK613136)	86.5	87	99.7	99.7	ID	99.7	91.9	91.9	84.8	84.8	85.1	84.8	85.1	93.8	93.8	94.1	91.8	91.8	84.1	84.1	84.1	90.3	90.3	90.6	91.4	94.9	94.6	94.6	93.5	94.6	92.7	93.5	93.3
Ixodes canisuga Romania (KY962060)	86.8	87.3	100	100	99.7	ID	92.2	92.2	85.1	85.1	85.3	85.1	85.3	94.1	94	94.3	92	92	84.4	84.4	84.4	90.6	90.6	90.9	91.6	95.1	94.9	94.9	93.8	94.9	93	93.8	93.5
Ixodes fujitai Japan (LC769934)	84.1	83.9	92.2	92.2	91.9	92.2	ID	99.5	84	84	84.3	84	84.3	89.8	89.8	90.1	91.2	91.2	82.3	82.3	82.3	89	90.6	89	90.1	91.7	91.7	91.7	91.4	91.7	89.2	91.4	92.5
Ixodes fujitai Japan (LC769955)	83.6	83.3	92.2	92.2	91.9	92.2	99.5	ID	84	84	84.3	84	84.3	89.8	89.8	90.1	91.2	91.2	81.8	81.8	81.8	89	90.6	89	90.1	91.7	91.7	91.7	91.4	91.7	89.2	91.4	92.5
Ixodes hexagonus USA (NC 002010)	85.9	85.9	85.1	85.1	84.8	85.1	84	84	ID	99.5	99.7	99.5	99.7	83	82.9	83	83.9	83.9	82.1	82.1	82.1	83.8	84.3	83.8	84.8	84.5	84.8	84.8	84.5	84.8	83.5	84.5	84.1
Ixodes hexagonus Austria (KY962058)	85.9	85.9	85.1	85.1	84.8	85.1	84	84	99.5	ID	99.7	100	99.7	83	82.9	83	83.9	83.9	82.1	82.1	82.1	83.8	84.3	83.8	84.8	84.5	84.8	84.8	84.5	84.8	84	84.5	84.1
Ixodes hexagonus Croatia (KY962076)	86.2	86.2	85.3	85.3	85.1	85.3	84.3	84.3	99.7	99.7	ID	99.7	100	83.2	83.2	83.2	84.2	84.2	82.4	82.4	82.4	84.1	84.6	84.1	85.1	84.8	85.1	85.1	84.8	85.1	83.7	84.8	84.4
Ixodes hexagonus Germany (KY962070)	85.9	85.9	85.1	85.1	84.8	85.1	84	84	99.5	100	99.7	ID	99.7	83	82.9	83	83.9	83.9	82.1	82.1	82.1	83.8	84.3	83.8	84.8	84.5	84.8	84.8	84.5	84.8	84	84.5	84.1
Ixodes hexagonus Romania (KY962063)	86.2	86.2	85.3	85.3	85.1	85.3	84.3	84.3	99.7	99.7	100	99.7	ID	83.2	83.2	83.2	84.2	84.2	82.4	82.4	82.4	84.1	84.6	84.1	85.1	84.8	85.1	85.1	84.8	85.1	83.7	84.8	84.4
Ixodes lividus Belgium (KJ414461)	84.4	84.4	94.1	94.1	93.8	94.1	89.8	89.8	83	83	83.2	83	83.2	ID	99.5	99.7	90.5	90.5	83.1	83.1	83.1	87.2	88.2	87.4	88.4	92.4	92.2	92.2	91.1	92.2	90	91.1	91.9
Ixodes lividus Czech Republic (KX159446)	84.9	84.9	94	94	93.8	94	89.8	89.8	82.9	82.9	83.2	82.9	83.2	99.5	ID	99.2	90.4	90.4	83.1	83.1	83.1	87.1	88.2	87.4	88.4	93	92.7	92.7	91.6	92.7	90.5	91.6	91.4
Ixodes lividus Lithuania (KT715821)	84.4	84.4	94.3	94.3	94.1	94.3	90.1	90.1	83	83	83.2	83	83.2	99.7	99.2	ID	90.7	90.7	83.3	83.3	83.3	87.2	88.5	87.4	88.7	92.7	92.4	92.4	91.4	92.4	90.3	91.4	92.2
Ixodes nipponrhinolophi Japan (LC769935)	85.1	85.4	92	92	91.8	92	91.2	91.2	83.9	83.9	84.2	83.9	84.2	90.5	90.4	90.7	ID	99.7	81.2	81.2	81.2	87.2	88.8	87.2	88.3	90.4	90.7	90.7	89.9	90.7	88	89.9	90.2
Ixodes nipponrhinolophi Japan (LC769946)	85.4	85.6	92	92	91.8	92	91.2	91.2	83.9	83.9	84.2	83.9	84.2	90.5	90.4	90.7	99.7	ID	81.4	81.4	81.4	87.5	89.1	87.5	88.6	90.4	90.7	90.7	89.9	90.7	88	89.9	90.2
Ixodes persulcatus China (PX067722)	87.6	86.8	84.4	84.4	84.1	84.4	82.3	81.8	82.1	82.1	82.4	82.1	82.4	83.1	83.1	83.3	81.2	81.4	ID	100	100	83.2	82.3	83.2	82.3	82.8	83.1	83.1	82.5	83.1	82	82.5	83.1
Ixodes persulcatus Japan (OL741748)	87.6	86.8	84.4	84.4	84.1	84.4	82.3	81.8	82.1	82.1	82.4	82.1	82.4	83.1	83.1	83.3	81.2	81.4	100	ID	100	83.2	82.3	83.2	82.3	82.8	83.1	83.1	82.5	83.1	82	82.5	83.1
Ixodes persulcatus Sweden (KX384801)	87.6	86.8	84.4	84.4	84.1	84.4	82.3	81.8	82.1	82.1	82.4	82.1	82.4	83.1	83.1	83.3	81.2	81.4	100	100	ID	83.2	82.3	83.2	82.3	82.8	83.1	83.1	82.5	83.1	82	82.5	83.1
Ixodes simplex China (OM368260)	86.1	85.5	90.6	90.6	90.3	90.6	89	89	83.8	83.8	84.1	83.8	84.1	87.2	87.1	87.2	87.2	87.5	83.2	83.2	83.2	ID	93.8	99.7	93.8	89	88.7	88.7	88.5	88.7	87.7	88.5	90.1
Ixodes simplex Hungary (KM455970)	86	85.5	90.6	90.6	90.3	90.6	90.6	90.6	84.3	84.3	84.6	84.3	84.6	88.2	88.2	88.5	88.8	89.1	82.3	82.3	82.3	93.8	ID	93.6	98.4	90.1	89.8	89.8	88.4	89.8	88.7	88.4	90.9
Ixodes simplex Japan (AB901140)	86.3	85.8	90.9	90.9	90.6	90.9	89	89	83.8	83.8	84.1	83.8	84.1	87.4	87.4	87.4	87.2	87.5	83.2	83.2	83.2	99.7	93.6	ID	94.1	89.2	89	89	88.7	89	87.9	88.7	90.3
Ixodes simplex South Africa (KY457532)	86.3	85.7	91.6	91.6	91.4	91.6	90.1	90.1	84.8	84.8	85.1	84.8	85.1	88.4	88.4	88.7	88.3	88.6	82.3	82.3	82.3	93.8	98.4	94.1	ID	90.8	90.6	90.6	89.2	90.6	89.5	89.2	91.1
Ixodes vespertilionis France (KR902772)	85.4	85.4	95.1	95.1	94.9	95.1	91.7	91.7	84.5	84.5	84.8	84.5	84.8	92.4	93	92.7	90.4	90.4	82.8	82.8	82.8	89	90.1	89.2	90.8	ID	99.7	99.7	97.8	99.7	96.2	97.8	92.7
Ixodes vespertilionis Hungary (KM455966)	85.1	85.1	94.9	94.9	94.6	94.9	91.7	91.7	84.8	84.8	85.1	84.8	85.1	92.2	92.7	92.4	90.7	90.7	83.1	83.1	83.1	88.7	89.8	89	90.6	99.7	ID	100	98.1	100	96.5	98.1	92.5
Ixodes vespertilionis Hungary (OL795953)	85.1	85.1	94.9	94.9	94.6	94.9	91.7	91.7	84.8	84.8	85.1	84.8	85.1	92.2	92.7	92.4	90.7	90.7	83.1	83.1	83.1	88.7	89.8	89	90.6	99.7	100	ID	98.1	100	96.5	98.1	92.5
Ixodes vespertilionis Spain (KR902773)	83.8	83.8	93.8	93.8	93.5	93.8	91.4	91.4	84.5	84.5	84.8	84.5	84.8	91.1	91.6	91.4	89.9	89.9	82.5	82.5	82.5	88.5	88.4	88.7	89.2	97.8	98.1	98.1	ID	98.1	95.7	100	91.4
Ixodes vespertilionis Turkey (ON540350)	85.1	85.1	94.9	94.9	94.6	94.9	91.7	91.7	84.8	84.8	85.1	84.8	85.1	92.2	92.7	92.4	90.7	90.7	83.1	83.1	83.1	88.7	89.8	89	90.6	99.7	100	100	98.1	ID	96.5	98.1	92.5
Ixodes vespertilionis Turkey (PV113064)	83.8	83.8	93	93	92.7	93	89.2	89.2	83.5	84	83.7	84	83.7	90	90.5	90.3	88	88	82	82	82	87.7	88.7	87.9	89.5	96.2	96.5	96.5	95.7	96.5	ID	95.7	91.9
Ixodes vespertilionis USA (U95910)	83.8	83.8	93.8	93.8	93.5	93.8	91.4	91.4	84.5	84.5	84.8	84.5	84.8	91.1	91.6	91.4	89.9	89.9	82.5	82.5	82.5	88.5	88.4	88.7	89.2	97.8	98.1	98.1	100	98.1	95.7	ID	91.4
***Ixodes ariadnae* Slovakia (PX474683)**	85.5	84.9	93.5	93.5	93.3	93.5	92.5	92.5	84.1	84.1	84.4	84.1	84.4	91.9	91.4	92.2	90.2	90.2	83.1	83.1	83.1	90.1	90.9	90.3	91.1	92.7	92.5	92.5	91.4	92.5	91.9	91.4	ID

80	85	90	95	100
